# Randomized Clinical Trial of Surgical vs. Percutaneous vs. Hybrid Revascularization in Multivessel Coronary Artery Disease: Residual Myocardial Ischemia and Clinical Outcomes at One Year—Hybrid coronary REvascularization Versus Stenting or Surgery (HREVS)

**DOI:** 10.1155/2020/5458064

**Published:** 2020-01-03

**Authors:** Vladimir Ganyukov, Nikita Kochergin, Aleksandr Shilov, Roman Tarasov, Jan Skupien, Wojciech Szot, Aleksandr Kokov, Vadim Popov, Kirill Kozyrin, Olga Barbarash, Leonid Barbarash, Piotr Musialek

**Affiliations:** ^1^Federal State Budgetary Institution “Research Institute for Complex Issues of Cardiovascular Diseases”, Kemerovo, Russia; ^2^Jagiellonian University School of Medicine, Krakow, Poland; ^3^Dept. of Nuclear Medicine, John Paul II Hospital, Krakow, Poland; ^4^Federal State Budgetary Institution A. V. Vishnevsky Institute of Surgery, Moscow, Russia; ^5^Jagiellonian University Dept. of Cardiac & Vascular Diseases, John Paul II Hospital, Krakow, Poland

## Abstract

**Aim:**

Optimal revascularization strategy in multivessel (MV) coronary artery disease (CAD) eligible for percutaneous management (PCI) and surgery remains unresolved. We evaluated, in a randomized clinical trial, residual myocardial ischemia (RI) and clinical outcomes of MV-CAD revascularization using coronary artery bypass grafting (CABG), hybrid coronary revascularization (HCR), or MV-PCI.

**Methods:**

Consecutive MV-CAD patients (*n* = 155) were randomized (1 : 1 : 1) to conventional CABG (LIMA-LAD plus venous grafts) or HCR (MIDCAB LIMA-LAD followed by PCI for remaining vessels) or MV-PCI (everolimus-eluting CoCr stents) under Heart Team agreement on equal technical and clinical feasibility of each strategy. SPECT at 12 months (primary endpoint of RI that the trial was powered for; a measure of revascularization midterm efficacy and an independent predictor of long-term prognosis) preceded routine angiographic control.

**Results:**

Data are given, respectively, for the CABG, HCR, and MV-PCI arms. Incomplete revascularization rate was 8.0% vs. 7.7% vs. 5.7% (*p*=0.71). Hospital stay was 13.8 vs. 13.5 vs. 4.5 days (*p* < 0.001), and sick-leave duration was 23 vs. 16 vs. 8 weeks (*p* < 0.001). At 12 months, RI was 5 (2, 9)% vs. 5 (3, 7)% vs. 6 (3, 10)% (median; Q1, Q3) with noninferiority *p* values of 0.0006 (HCR vs. CABG) and 0.016 (MV-PCI vs. CABG). Rates of angiographic graft stenosis/occlusion or in-segment restenosis were 20.4% vs. 8.2% vs. 5.9% (*p*=0.05). Clinical target vessel/graft failure occurred in 12.0% vs. 11.5% vs. 11.3% (*p*=0.62). Major adverse cardiac and cerebral event (MACCE) rate was similar (12% vs. 13.4% vs. 13.2%; *p*=0.83).

**Conclusion:**

In this first randomized controlled study comparing CABG, HCR, and MV-PCI, residual myocardial ischemia and MACCE were similar at 12 months. There was no midterm indication of any added value of HCR. Hospital stay and sick-leave duration were shortest with MV-PCI. While longer-term follow-up is warranted, these findings may impact patient and physician choices and healthcare resources utilization. This trial is registered with NCT01699048.

## 1. Introduction

The optimal revascularization strategy in multivessel coronary artery disease (MV-CAD) remains unresolved. The longevity of the left internal mammary artery (LIMA) to LAD graft contributes substantially to the survival benefit of coronary artery bypass grafting (CABG), while the major benefit of multivessel percutaneous intervention (PCI) is lesser invasiveness [1]. With contemporary (2^nd^ generation) drug-eluting stents (DES), combined restenosis and thrombosis rate is lower than saphenous vein graft failure [2, 3]. Excellent outcomes of the LIMA-LAD graft and favourable outcomes of contemporary DES are the basis for active consideration of hybrid coronary revascularization (HCR; LIMA-LAD plus DES-PCI for remaining vessel/s) as the “third” contemporary revascularization approach to treat patients with MV-CAD [1, 2, 4–9]. HCR is defined as a planned intervention combining cardiac surgery with a catheter-based intervention performed within a predefined time [2, 4]. HCR employing a combination of a minimally invasive LIMA-LAD graft procedure with PCI using DES to non-LAD vessels is receiving increasing attention [10–12] but it has not yet been evaluated in a clinical trial involving the two leading MV-CAD revascularization modalities, CABG and PCI [1].

Single-photon emission computed tomography (SPECT) myocardial perfusion imaging, with percent ischemic myocardium (residual ischemia, RI, calculated by subtracting the rest from stress total perfusion defect) is not only an objective method to compare the outcome of coronary revascularization but also there is a direct proportional relationship between RI extent and prognosis [13]. This makes RI an attractive quantitative endpoint with an evidenced relationship to long-term cardiovascular events.

HREVS (Hybrid coronary REvascularization Versus Stenting or Surgery) was designed as the first randomized controlled study to assess safety and efficacy of the three contemporary MV-CAD revascularization modalities employing RI as the quantifiable primary endpoint.

## 2. Materials and Methods

### 2.1. Study Design

The HREVS trial was a prospective, randomized, open label, multiarm parallel-group, safety and efficacy study. The study protocol was approved by the local ethics committee and it complies with the Declaration of Helsinki. Written informed consent was obtained from all study participants. All consecutive patients with angiography-confirmed MV-CAD involving LAD and a significant (≥70% diameter stenosis, DS, on quantitative coronary angiography, QCA) lesion in at least one major non-LAD epicardial vessel of ≥2.5 mm in diameter, amenable to PCI and CABG and HCR, were screened by a local Heart Team (HT). Lesions of 50–70% DS were subjected to functional evaluation and were considered the study target lesions (i.e, were labelled for revascularization) if lesion-related myocardial ischemia was present on functional testing (fractional flow reserve, FFR, or SPECT stress imaging). A list of inclusion/exclusion criteria is provided in Appendix A. HT evaluated all the inclusion/exclusion criteria with a particular attention given to equal angiographic and clinical feasibility to perform CABG or HCR or PCI. All consecutive HT-cleared patients were offered participation in the study. Those enrolled in the study (155 out of 204 HT-identified eligible patients over 31 months; NB. 24%, subjects refused random allocation of the treatment strategy) were randomized (external randomization on a 1 : 1 : 1 ratio) to standard surgical revascularization (CABG with LIMA to LAD and venous grafts to other vessels as a standard of reference) or HCR (MIDCAB LIMA-LAD plus PCI for non-LAD vessel/s) or MV-PCI until all study arms reached 50 subjects. In HCR, MIDCAB LIMA-LAD was always a first-stage procedure; PCI for the remaining vessels was performed within 3 days from surgery. All PCIs employed everolimus-eluting CoCr stents (Xience, Abbott Vascular, Abbott Park, Illinois, USA) as a standard of reference DES [3]. Treatment was within 7 days from randomization.

Prior to this trial, the study team had built experience in performing the trial procedures (yearly volume of over 700 CABG with over 100 LIMA-LAD MIDCAB, and over 800 PCIs). The study was sponsored by the Russian Academy of Sciences (RAS 056-2013-0012). The sponsor had no influence on the study protocol, management, or data analysis.

Primary follow-up point was at 12 ± 1 months. Analysis was intention to treat (ITT).

### 2.2. Primary Endpoint

Primary endpoint was residual ischemia at 12 ± 1 months by SPECT, with protocol-mandated SPECT preceding (by 3–7 days) the protocol-mandated angiographic control. SPECT protocol and data analysis were according to those in the COURAGE Nuclear substudy [13]. In brief, patients underwent a 1- or 2-day protocol with rest ^99m^Tc sestamibi combined with stress ^99m^Tc sestamibi. The percent ischemic myocardium was calculated by subtracting the rest from the stress total perfusion defect. SPECT analysis was performed in a blinded fashion in an external nuclear medicine laboratory (Dept. of Nuclear Medicine, John Paul II Hospital, Krakow, Poland) using Quantitative Perfusion SPECT software (Cedars-Sinai Medical Center, Los Angeles, CA, USA).

### 2.3. Secondary Endpoints

Secondary endpoints included (i) incomplete revascularization (on a lesion- and patient-basis), (ii) MACCE (a composite of all-cause death, myocardial infarction (MI), stroke, and clinically driven target vessel revascularization) at 30 days and 12 months, (iii) length of hospitalization, use of postdischarge inpatient institutional rehabilitation program, and sick-leave duration, and (iv) target vessel (TV) or graft failure (TFV; a composite of cardiac death, TV-MI, and clinically driven target vessel revascularization, TVR) at 12 months after randomization. For endpoint definitions, see Appendix B.

Angiographic analysis was verified, inclusive of blinded analysis of baseline angiograms and SYNTAX score calculation, by an external laboratory using complete angiographic data. Clinical events were adjudicated by an external clinical events committee that had access to patient source data.

### 2.4. Revascularization, Pharmacological Treatment, and Angiographic Follow-Up

For the CABG procedure, venous revascularization (except LIMA to LAD) was performed according to routine practice in the study centre. In the HCR group, MIDCAB was always a first-stage procedure and it was followed, within 3 days, by PCI.

Aspirin was prescribed before revascularization for all study patients and it was continued indefinitely. For PCI (including HCR PCI), UFH was used (i.v. bolus of 100 IU per kilogram body weight followed by adjustment according to target-activated clotting time of 250 to 300 seconds). Antiplatelet regimen included clopidogrel routinely (loading dose of 300 mg at the time of PCI unless used in advance; then 75 mg daily, recommended duration of treatment 12 months) and aspirin (75 mg once daily) indefinitely.

Complete revascularization was defined as successful revascularization, by means of either surgery (bypassing) or PCI (stenting), of all HT-determined target lesions. Incomplete revascularization was evaluated on a lesion- and patient-basis.

Any potential angiography (±PCI) performed for clinical reason(s) prior to the study primary follow-up point had no influence on adhering to protocol-mandated angiographic control at 12 ± 1 months.

Postprocedure lifestyle modification and medication regimen were according to guideline recommendations.

### 2.5. Statistical Analysis

Central database management and statistical analysis were external. For the primary endpoint, RI differences between the study arms (with CABG taken as reference) were tested against a prespecified noninferiority margin of 4.2 percentage points based on literature data of the clinically relevant threshold of RI difference (see Appendix D). *p* values <0.025 were considered significant to adjust for two comparisons of the primary endpoint. In addition, comparison of each vs. each revascularization method was performed with the RI differences between the study arms (expressed as positive values) tested against a prespecified noninferiority margin of 4.2 percentage points and the *p* value spending function to calculate overall type I error rate. Thus, the additional analysis was deliberately performed in absence of defining a reference method of MVD revascularization. To control type I error rate, we calculated the overall type I error rate in three pairwise comparisons of the treatment arms from the formula (1 − *p*_total_)=(1 − *p*_1_) × (1 − *p*_2_) × (1 − *p*_3_), and *p*_total_ was considered statistically significant when <0.05.

For comparisons of secondary endpoints and clinical characteristics, nominal *p* values <0.05 were considered significant. Power calculations are described in Appendix C.

## 3. Results

One hundred and fifty-five consecutive patients with MV-CAD, who met the inclusion/exclusion criteria, were randomized to CABG (*n* = 50), HCR (*n* = 52), or MV-PCI (*n* = 53) following HT agreement on equal technical and clinical feasibility of each of the 3 coronary revascularization modes. Baseline clinical and procedural characteristics of the study patients are shown in Table 1. Of note, the mean age was 62 ± 7 years, and the majority of the patients were males (71.6%). More than one half of the patients had a prior MI (55.5%). Left ventricular ejection fraction (LVEF) was 54.5 ± 8.0%. One half of the study patients had 2-vessel disease (50.3%), whereas ≥3 vessel CAD was present in the other half (49.7%). Mean angiographic SYNTAX Score was 19.4 ± 2.9, whereas EuroScore II was 1.71 ± 0.76. Baseline characteristics were similar between the study arms (Table 1). Numbers of diseased vessels and index lesions as well as CR stents and grafts are given in Table 1.

The HCR patients, except 5 (9.8%) who required conversion to CABG (Table 2), had per-protocol PCI within 3 days (in most cases at 24–48 h) after performing MIDCAB LIMA-LAD anastomosis that was always the first stage of HCR. The reasons for HCR conversion to conventional on-pump CABG with median sternotomy were either technical surgical (*n* = 2) or there was hemodynamic instability following LIMA-LAD grafting that required other lesions' revascularization on an ad hoc basis (*n* = 3). All other patients in the HCR group (47/52) continued to the PCI stage of HCR and underwent, at that point, angiographic LIMA control. This showed LIMA thrombotic occlusion in 1 case (2.1%) resolved by PCI of the native artery, LAD, using the study DES. Thus, the HCR LIMA immediate patency rate was 46/47 (97.9%). Patent LIMAs and native LADs showed TIMI-3 flow in absence of any anastomosis stenosis >50% DS that might warrant considering a need for intervention.

Target coronary revascularization was incomplete, on a per-lesion basis, in 3.7% (5/136) in the CABG group versus 2.7% (4/149) and 2.1% (3/146) lesions in the HCR and PCI groups, respectively (*p*=0.71). On a per-patient basis, incomplete revascularization rate was 8.0% (4/50) vs. 7.7% (4/52) vs. 5.7% (3/53) patients (*p*=0.86) (Table 1).

Bleeding was more prevalent, and it was greater (for BARC evaluation see Table 2), in the study arms involving surgery. There were 4 transfusions in CABG (8.0%), 2 in HCR (3.9%), and none in the MV-PCI arm (*p*=0.066). There was one death ≤30 days that occurred in the HCR group (the patient experienced periprocedural MI and stroke that led to death).

The length of hospitalization, use of inpatient rehabilitation, and sick-leave duration were higher with surgery (CABG or HCR), with hospitalization length and institutional rehabilitation similar in the CABG and HCR arms (Table 2). Thirty-day MACCE rate was 8% vs. 5.8% vs. 3.8% (respectively, CABG, HCR, and PCI, *p*=0.37), and it was driven mainly by periprocedural MI (Table 2).

At 12 ± 1 months, all alive patients (*n* = 149) underwent protocol-mandated SPECT that was followed by protocol-mandated control angiography. None of the 5 patients with repeat revascularization prior to the primary follow-up at 12 ± 1 months (Table 1) was alive at the point of protocol-mandated SPECT (5 out of 6 deaths before 12 ± 1 months occurred in patients with repeat revascularization).

**Figure 1:**
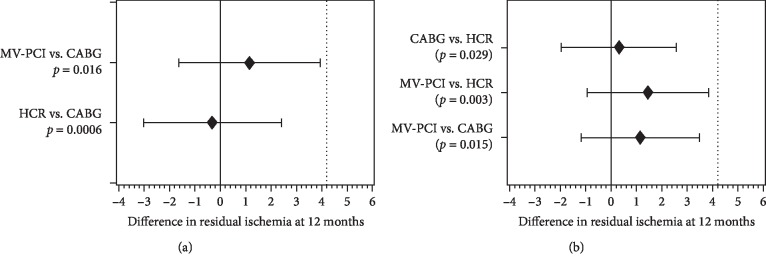
Noninferiority analysis for the SPECT-based residual ischemia at 12 months in the three treatment arms with CABG as a reference method (a) and assuming no single reference method (b). Point estimates and 90% confidence intervals for the differences in RI between treatment modalities are shown with solid vertical gridline indicating the null difference and interrupted vertical gridline indicating the noninferiority margin of 4.2 percentage points. (a) Respective *p* values are for noninferiority of MV-PCI vs CABG and HCR vs. CABG. To adjust for two comparisons with CABG as the reference *p* values were considered statistically significant when <0.025. (b) *p* values are for pairwise noninferiority tests with 95% one-sided confidence intervals. Overall *p* for noninferiority is 0.046.

**Figure 2:**
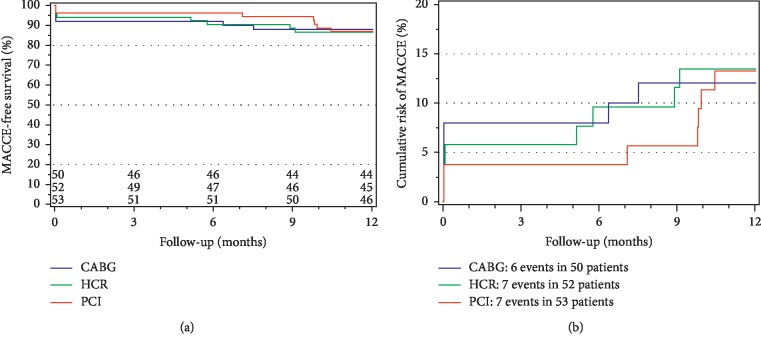
MACCE-free survival (a) and cumulative risk of MACCE (b) during 12-month follow-up according to the treatment arm. Panel A shows MACCE-free survival, whereas the cumulative risk of MACCE is depicted in Panel B. Numbers of patients at risk are shown above the horizontal axis in panel A. Pairwise comparisons of treatment arms with Cox proportional hazards model are shown at the bottom of panel B. MACCE—Major Adverse Cardiac or Cerebral Event.

Median RI at 12 months was mild in all study arms; 5 (2, 9)% vs. 5 (3, 7)% vs. 6 (3, 10)% (median; Q1, Q3) with the noninferiority *p* values of 0.0006 (HCR vs. CABG) and 0.016 (MV-PCI vs. CABG). Between-group differences were significantly smaller than the prespecified noninferiority margin of 4.2% and the trial met its primary endpoint of noninferiority (Figure 1). The ITT-based conclusion was the same when patients with conversion from HCR were excluded (per protocol analysis) and when the patients with conversion from HCR were reclassified to the CABG group (per treatment analysis). There were no differences in the primary endpoint in patients with 2-vessel disease vs. >2-vessel disease. There were also no differences according to SYNTAX score. Proportion of patients with RI>5% was similar in all three treatment modalities (CABG 20/49, 40.8%; HCR 21/49; 42.9%; MV-PCI 26/51; 51.0%, *p*=0.56). As shown in Figure 2, the three treatment modalities were associated with a similar freedom from MACCE at 12 months.

Angiographic control at 12 months demonstrated 9 SVGs and 1 LIMA stenosis/occlusion in the CABG group (10/49, 20.4%), 3 LIMA stenoses/occlusions and 1 in-segment restenosis in the HCR group (4/49, 8.2%), and 3 in-segment restenoses in the PCI group (3/51, 5.9%); *p*=0.05. Twelve-month TV or graft failure (composite of cardiac death, TV-MI, and clinically driven TVR) was 12.0% (CABG) versus 11.5% (HCR) versus 11.3% (PCI) (*p*=0.99). Angiography-driven revascularization was performed in 1 of 50 (2.0%) CABG patients versus 12/105 (11.4%) subjects with any PCI at baseline (combined HCR plus MV-PCI arm) (*p*=0.062). As shown in Table 2, total TVR rate (sum of clinically driven and control angiography-driven) at 12 months numerically favoured CABG, with 4.0% (CABG) versus 13.5% (HCR) versus 17.0% (PCI) (*p*=0.095), and 4.0% in the CABG arm (2/50) but 15.2% (16/105) in the combined HCR plus MV-PCI cohort (*p*=0.058; for individual group data, see Table 2).

## 4. Discussion

The primary endpoint of this first randomized controlled study, comparing conventional CABG, MV-PCI using 2^nd^ generation standard-of-reference DES, and HCR in patients with MV-CAD amenable to treatment with any of the three guideline-accepted modalities, was SPECT-determined RI at 12 months. Based on the noninferiority margin of the trial, the three strategies were similar after 12 months in terms of RI (Figure 1) that is an established measure of CR efficacy and a predictor of long-term prognosis. Other important findings, with potential relation to healthcare resources' utilization, are shorter hospital stay, less need to use institutional in-patient rehabilitation, and shorter sick-leave duration with the percutaneous route of coronary revascularization in MV-CAD. Although underpowered for clinical events, HREVS suggests similar 12-month MACCE rates with all three treatment strategies (Figure 2), a finding that requires confirmation in a larger multicentre study.

The primary focus of HREVS is RI at 12 months by SPECT. Myocardial perfusion SPECT imaging is not only an objective method to compare the outcome of coronary revascularization but also there is a direct proportional relationship between the extent of RI and prognosis [14, 15]. Taking into account that the groups were randomized, comparable in their basic characteristics, and the fact that the groups received high level of complete revascularization (92% vs. 92.3% vs. 94.3%), it can be concluded that within the assumed noninferiority margin, the three strategies occurred similar with respect to RI at 12 months. This main result is broadly consistent with the analysis of the secondary endpoints (Table 2, Figure 2).

The two typically applied techniques for MV-CAD interventional management, CABG and MV-PCI, have clinically relevant disadvantages that include the invasiveness of CABG and the increased risk of repeat revascularization with PCI [2, 16]. The optimal revascularization approach would thus need to combine a decreased invasiveness plus low risk of perioperative complications and an increased durability and survival. A combination of a minimally invasive LIMA-LAD graft procedure with PCI using DES to non-LAD vessels eliminates aortic manipulation and extracorporeal circulation, resulting in a potential to decrease the risk of perioperative complications [1, 4, 11, 12, 17]. Thus, the “third” revascularization strategy—HCR—might have potential advantages beyond PCI and CABG alone [1, 4–12, 18, 19]. Although HCR was first introduced over 20 years ago [4], today the potential of this strategy in MV-CAD patients appears neither sufficiently determined [1, 2, 10] nor fully utilized [9, 19]. Some fundamental HCR concerns include the complexity of patient logistics; the presence of surgical and endovascular stage (with “naturally” incomplete revascularization at the HCR first-stage); the timing of antiplatelet therapy, optimal timing of the HCR stages; and technical aspects of the surgical intervention (access site, and the role of thoracoscopic or robotic approaches) [5–11].

Thus far, HCR outcomes have been compared mostly with standard CABG [1, 6, 8], and included only one randomized study that, however, did not have a percutaneous treatment arm [6]. Another observational study compared conventional CABG to MV-PCI [18]. Retrospective series and meta-analyses have reported low mortality rates (0% to 2%) and event-free survival rates of 83% to 92% for HCR at 6 to 12 months of follow-up and similar outcomes of HCR in comparison with standard revascularization options [5, 8]. In the single randomized trial of HCR vs. conventional CABG, the HCR arm demonstrated, at 12 months, similar to CABG cumulative occurrence of major adverse cardiac events [6]. In that study, 6.1% HCR patients required conversion to standard CABG [6], a result broadly consistent with our present findings (Table 2).

HREVS is the first randomized controlled study comparing outcomes of the three guideline-accepted treatment strategies in MV-CAD patients. In HREVS, the HCR patients underwent two-stage revascularization with MIDCAB first, followed by PCI using the second-generation everolimus-eluting stents. The use of a standard-of-reference [3, 20, 21] 2^nd^ generation DES in HREVS, the device that showed some of the best results in the interventional treatment of CAD patients, suggests that HREVS patients were offered a maximized benefit from the choice of stent in the endovascular arm and in the HCR arm.

Prior work indicated that MIDCAB, when compared to conventional sternotomy CABG, results in less surgical trauma, decreased risk of bleeding and infectious complications, and may shorten the length of hospitalization [4, 11, 12]. The latter, however, is not supported by our findings, a result that may be partly driven (note ITT analysis) by the conversion rate (9.8%) from HCR to CABG in HREVS (Table 2).

In the HREVS HCR arm, PCI was performed within 3 days after surgery (in majority of patients, within 24–48 h after surgery). This allowed consistency of hemostasis in absence of DAPT at the time of surgery (the patients were operated on aspirin and loaded with clopidogrel at the time of PCI) as well as angiographic control of the LIMA-to-LAD graft during the endovascular stage. Lack of *ad hoc* total revascularization in the HCR group with LIMA-to-LAD MIDCAB, however, was associated with hemodynamic instability and myocardial ischemia in 3 patients who required conversion to sternotomy to perform ad hoc revascularization of the remaining lesions by CABG.

Although HREVS will continue to monitor its study participants up to 5 years, a larger multicentre study involving HCR along CABG and MV-PCI would be required to determine, by clinical outcomes, the optimal interventional treatment strategy in MV-CAD. This is relevant also because evidence is accumulating that using multiple arterial grafts in CABG may be associated with improved clinical outcomes when compared to either conventional CABG with LIMA to LAD and SVGs to other vessels [22], CABG using bilateral mammary arteries plus SVGs [23], or to MV-PCI [24]. Although HREVS indicates no significant differences in 12-month TVF between the three treatment modalities, the angiographic control at 1 year suggested sizable differences in graft stenosis/occlusion or in-segment restenosis rates (20.4% vs. 8.2% vs. 5.9% for CABG vs. HCR vs. MV-PCI; *p*=0.05) that may affect long-term outcomes [25]. Whether these differential 12-month anatomical revascularization results affect longer-term clinical outcomes [25] remains to be established.

Importantly, the lower rates of institutional rehabilitation use in the MV-PCI arm are not necessarily beneficial because the patients who opt not to use the institutional rehabilitation services might benefit from those.

The prevalence and extent of bleeding with (any) surgery (Table 2) should serve as an important consideration point in support of PCI rather than CABG or HCR in some (if not most of) moderate SYNTAX patients.

A recently funded US National Institutes of Health (NIH) Hybrid Coronary Revascularization Trial (HCR, NCT03089398), a 2354 patient study with 5-year follow-up, might be able to overcome only some of the HREVS limitations because the NIH HCR Trial compares HCR vs. MV-PCI in absence of the CABG arm.

### 4.1. Limitations

This study has several limitations as listed below.

- HREVS was not powered for clinical events, although the center enrolment rate and volume exceeded by over 10-fold typical yearly contributions in the pivotal BEST Trial comparing MV-PCI with CABG that was itself underpowered due to an insufficient and slow enrolment that included only 20% of the eligible patients [20].
- Recruitment challenges were related not only to the fundamental requirement of equal technical and clinical feasibility of either of the tested strategies but also to the patient's (and family's) natural gravitation towards less invasive treatment (evidenced by nearly 1 in 4 refusal rate to random treatment allocation due to PCI preference); thus, overall recruitment rate in HREVS was >75%.
- HREVS did not evaluate quality of life, an area where clinically relevant differences might exist, consistent with our findings on the sick-leave duration and time-to-return to work, favouring less invasive treatment strategies.
- Any generalizability of the findings needs to take into account the moderate MVD angiographic complexity in this study (reflecting the requirement of technical feasibility of CABG, HCR, and MV-PCI; thus, the need to exclude the left main coronary artery stenosis not amenable to HCR, severely calcific lesions, complex bifurcations, or chronic total occlusion that may favour surgery) and the particular sequence and timing of HCR procedures as per the HREVS protocol.

### 4.2. Strengths

Fundamental strengths of HREVS include the following:

- Use of quantifiable primary endpoint of RI at 12 months that is independently predictive, in a gradient manner, of cardiac death or MI [13–15, 26], and the trial appropriate power for noninferiority comparison of the 3 treatment modalities [13, 27].
- There were no identifiable clinical or angiographic differences between the patients who agreed to random treatment allocation in HREVS and entered the study versus those who were Heart Team–labelled as eligible for enrolment but did not accept random treatment allocation, in favour of generalizability of the findings to similar patients outside the trial.
- Mandatory angiographic control at 12 months is a particular strength of the present study as it verified the midterm anatomic quality of revascularization.
- HREVS results add importantly to the present knowledge in the context of (a) increasing penetration of percutaneous revascularization [28], (b) suggestions that optimized PCI might lead to CABG-like outcomes in MV-CAD [29], and (c) increasing Heart Team recommendations of first-line percutaneous approach [2, 28].
- Rather than generating hypotheses on the basis of historical comparative data [29], HREVS was a real-life randomized trial with multiarm parallel-group design.

## 5. Conclusion

In patients with MV-CAD amenable to CABG, HCR, and MV-PCI, the quantitative endpoint of residual myocardial ischemia at 12 months, which is predictive in a gradient manner of cardiac death and adverse cardiac events [13–15, 26], was similar with all three guideline-accepted revascularization strategies. MV-CAD PCI, using contemporary best-in-class drug-eluting stents, was associated with a shorter hospital stay, less inpatient rehabilitation, and shorter sick-leave duration than CABG or HCR.

While extended follow-up will determine longer-term outcomes from the present study, a larger-scale multicentre trial powered for clinical endpoints would be warranted. Nevertheless, any effective execution of such a large-scale study seems unlikely [20].

## Figures and Tables

**Table 1:** Baseline and procedural characteristics according to randomization arm^*∗*^.

Characteristic	CABG (*n* = 50)	HCR (*n* = 52)	PCI (*n* = 53)	*p*
Age (years)	61.3 ± 6.8	62.0 ± 7.4	61.7 ± 7.7	0.80
Male sex	70.0% (35)	75.0% (39)	69.8% (37)	0.90
Current smoking	50.0% (25)	46.1% (24)	47.2% (25)	0.92
Arterial hypertension	66.0% (33)	65.4% (34)	67.9% (36)	0.96
Diabetes mellitus	22.0% (11)	17.3% (9)	20.7% (11)	0.83
Chronic kidney disease	0% (0)	1.9% (1)	5.7% (3)	0.32
COPD/BA^†^	4.0% (2)	7.7% (4)	11.3% (6)	0.43
Previous MI^‡^	56.0% (28)	51.9% (27)	58.5% (31)	0.79
Prior stroke	6% (3)	7.7% (4)	5.7% (3)	0.92
Peripheral vascular disease	24.0% (12)	30.8% (16)	30.2% (16)	0.70
LVEF (%)^§^	54.0 ± 7.4	56.2 ± 6.3	53.3 ± 9.9	0.159
LVEF≤45%	12% (6)	5.8% (3)	20.8% (11)	0.070
EuroSCORE II^ǁ^	1.70 ± 0.76	1.71 ± 0.72	1.70 ± 0.79	1.0
Affected vessels:				
2	42.0% (21)	51.9% (27)	56.6% (30)
≥3	58.0% (29)	48.1% (25)	43.4% (23)	0.32
Affected vessels (mean)	2.7 ± 0.6	2.5 ± 0.6	2.5 ± 0.6	—
No. of index lesions				
2	42.0% (21)	36.5% (19)	50.9% (27)
3	44.0% (22)	42.3% (22)	30.2% (16)	
>3	14.0% (7)	21.2% (11)	18.9% (10)	0.35
No. of index lesions (mean)	2.7 ± 0.7	2.9 ± 0.8	2.7 ± 0.9	—
^¶^SYNTAX score	19.3 ± 3.0	19.4 ± 3.0	19.5 ± 2.7	0.91
No. of grafts				
1	0% (0)	90.4% (47)	—
2	46.0% (23)	5.8% (3)	—	
≥3	54.0% (27)	3.8% (2)	—	NA
Arterial grafts	37.8% (50)	77.6% (52)	—	NA
Venous grafts	62.2% (82)	22.4% (15)	—	NA
No. of grafts (mean)			—	—
No. of stents				
0	2.6 ± 0.7	1.1 ± 0.4
1	—	9.6% (5)	0	
2	—	48.1% (25)	0	
3 or more	—	32.7% (17)	51.9% (27)	NA
No. of stents (mean)	—	9.6% (5)	49.1% (26)	—
	—	1.5 ± 0.9	2.7 ± 0.9	
Incomplete TLR^Φ^ (per patient)	8.0% (4)	7.7% (4)	5.7% (3)	0.86
Incomplete TLR^Φ^ (per total number target lesions in study group)	3.7% (5/136)	2.7% (4/149)	2.1% (3/146)	0.71

Values are means ± SD or percentages (counts). Data are shown as per randomization (intention-to-treat population). CABG: coronary-artery bypass grafting; HCR: hybrid coronary revascularization; PCI: percutaneous coronary intervention. ^†^COPD/BA: chronic obstructive pulmonary disease/bronchial asthma. ^‡^MI: myocardial infarction. ^§^LVEF: left ventricular ejection fraction. ^ǁ^EuroSCORE II: The European System for Cardiac Operative Risk Evaluation (EuroSCORE); a clinical model for calculating the risk of death after cardiac surgery. ^¶^SYNTAX score: Synergy between PCI with Taxus and Cardiac Surgery (SYNTAX) score; an angiographic model for evaluating coronary artery disease extensiveness. ^Φ^TLR, target lesion revascularization, given per total number of lesions to be revascularized according to Heart Team recommendation.

**Table 2:** HREVS study endpoints according to randomization group.

Endpoint	CABG	HCR	PCI	*p* value
Primary endpoint at 12 months^*∗*^				
	*N* = 49	*N* = 49	*N* = 51	
RI (SPECT)	6.7 (4.6, 8.8)	6.4 (4.3, 8.5)	7.9 (5.9, 9.8)	0.45^*∗∗*^

Secondary endpoints at 12 months				
	*N* = 50	*N* = 52	*N* = 53	
MACCE (death/stroke/MI/clinically driven repeat revascularization)	12.0% (6)	13.4% (7)	13.2% (7)	0.83
Death	2.0% (1)	5.8% (3)	3.8% (2)	0.78
Stroke	0% (0)	3.8% (2)	0% (0)	0.21
MI	8% (4)	5.8% (3)	7.5% (4)	0.66
Clinically driven TVR	2.0% (1)	1.9% (1)	5.7% (3)	0.54
Angiography-driven TVR	2.0% (1)	11.5% (6)	11.3% (6)	0.139
Total TVR	4.0% (2)	13.5% (7)	17.0% (9)	0.095

Secondary endpoints at 30 days				
MACCE (death/stroke/MI/clinically driven repeat revascularization)	8% (4)	5.8% (3)	3.8% (2)	0.37
Death	0% (0)	1.9% (1)	0% (0)	0.66
Stroke	0% (0)	1.9% (1)	0% (0)	0.66
MI	8% (4)	5.8% (3)	3.8% (2)	0.37
Repeat revascularization	0% (0)	1.9% (1)	0% (0)	0.66
Conversion to CABG	NA	9.6% (5)	0	0.027

Bleeding				
BARC 0–1	80.0% (40)	80.8% (42)	98.1% (52)	
BARC 2	0% (0)	9.6% (5)	1.9% (1)	
BARC 3–4	20.0% (10)	9.6% (5)	0% (0)	0.001
Hospital stay (days)	13.8 (12.5, 15.1)	13.5 (12.2, 14.8)	4.5 (3.2, 5.8)	<0.001
Institutional rehabilitation	100% (49)	97.9% (48)	56.8% (29)	<0.001
Sick leave (weeks)	23 (21, 25)	16 (15, 18)	8 (6, 10)	<0.001

Data are presented as means (95% confidence interval) or percentages (counts). ^*∗*^Evaluable in patients alive at 12 ± 1 months. ^*∗∗*^*p*=0.046 on combined noninferiority analysis that the study was powered for (cf. Figure 1).

**Table 3:** Inclusion and exclusion criteria of the HREVS trial.

Inclusion criteria	Exclusion criteria
1. Male or female ≥18 years of age 2. II–IV Canadian Cardiovascular Society functional class of angina 3. Angiographically confirmed multivessel coronary artery diseases involving LAD, with lesions severity ≥70% diameter stenosis (DS) by quantitative coronary angiography (QCA), *or* 50–70% DS with functional evidence of ischemia by either FFR ≤0.80 or stress SPECT 4. At least 1 month after acute MI (in patients with history of MI) 5. Heart team-determined indication to coronary revascularization with equal feasibility to perform complete revascularization using either of the three methods (HCR, MVD-PCI, CABG) 6. Written informed consent for participation in the study, including random treatment allocation and compliance with study requirements inclusive of follow-up visits and 12 ± 1 month SPECT followed by control angiography	1. Acute coronary syndrome (ACS) 2. Any previous coronary revascularization (CABG, HCR, or PCI) 3. Presence of any condition or abnormality that in the opinion of the investigator would compromise the safety of the patient or the quality of the data 4. Pregnancy 5. Stenosis of the left main coronary artery requiring revascularization 6. Significant calcification or occlusion of a major coronary vessel 7. Left ventricle aneurysm or valvular heart disease requiring surgical management 8. Comorbidity associated with an increased procedural risk for any of the treatment strategies or other study procedures 9. Peripheral arterial disease with pain-free walking distance ≤50m 10. Life expectancy ≤5 years 11. Inability to comply with dual antiplatelet therapy (DAPT) 12. Inability to undergo follow-up procedures including long-term follow-up 13. Participation in another clinical study

## Data Availability

Clinical, imaging, and statistical data used to support the findings of this study are included within the article and within the supplementary information file (Appendix).

## Appendix

### A. HREVS Trial Inclusion/Exclusion Criteria

Table 3 shows the inclusion and exclusion criteria of the HREVS trial.

### B. Endpoint and Other Definitions

MI and stroke were defined according to international guidelines (1, 2); other definitions were according to the Academic Research Consortium (3). In brief, clinically driven TVR was defined as percutaneous revascularization or bypass of the target lesion or any segment of the epicardial coronary artery containing the target lesion or more proximal vessels that may have been traversed by the angioplasty guidewire during the index procedure, driven by ischemic symptoms presence or presence of other clinical abnormalities leading to an angiogram prior to the protocol-mandated point at 12 ± 1 months from randomization. Angiography-driven TVR was TVR resulting from performing protocol-required control angiography at 12±1 months. Target vessel (TV) or graft failure was defined as a composite of cardiac death, target vessel-MI, and clinically driven TVR of the artery containing the target lesion (i.e., one of the index lesions in a given patient) within 12 months after randomization (3).

### C. Power Calculations

Power calculations and statistical analysis are consistent with reference 4 and reference 5. With recruitment of 50 subjects per group, the study had 80% power to exclude with a noninferiority *t*-test with the 2.5% type I error rate (adjusted for two comparisons with the reference arm), the margin set at 4.2 percentage points, which we considered a reasonable minimum clinically significant difference. An absolute 4.2 percentage points difference is associated with an increased risk of MI, whereas 4.9 percentage points is cut-off for an increased risk of death (6, 7), and the generally accepted clinically significant ischemia interval is set at 5%, with 5% considered a clinically significant difference (8, 9). Based on literature data (8), the assumed standard deviation of percent ischemic myocardium was 7.

### D. Additional Information on Statistical Analysis and Study Conduct and Reporting

The continuous variables are presented as the mean ± SD, unless with skewed distribution, for which medians with quartiles are presented. The categorical variables are summarized as percent and count. Study endpoints were reported as means ± SD with 95% confidence intervals or as percentages, where applicable. Analysis was intention to treat. Continuous variables were compared using unbalanced ANOVA when distributions were approximately normal. Kruskal–Wallis test was used to compare medians of significantly skewed variables. Proportions were compared with the chi^2^ test or the Fisher exact test, as appropriate. A noninferiority analysis for the primary endpoint was performed using the t-distribution-based confidence intervals assuming a noninferiority margin of 4.2 RI percentage points. To adjust for two comparisons with CABG as the reference *p* values were considered statistically significant when <0.025. A 2-sided nominal *p* value <0.05 was considered statistically significant in secondary endpoint analysis. Product-limit survivor function estimate was used in MACCE analysis, and Cox proportional hazards model was applied to estimate hazard ratios of MACCE between study arms. Statistical analysis was performed with SAS 9.4 software (SAS Institute, Cary, NC, USA).

HREVS study conduct and reporting were consistent with the updated CONSORT 2010 guidelines [10].

### E. Appendix Literature

1. Thygesen K, Alpert JS, Jaffe AS, et al. “Fourth universal definition of myocardial infarction”. Circulation, vol. 138, no 20, pp. e618–e651, 2018.
2. Sacco RL, Kasner SE, Broderick JP, et al. “An updated definition of stroke for the 21st century: A statement for healthcare professionals from the American Heart Association/American Stroke Association”. Stroke, vol. 44, no 7, pp. 2064–89, 2013.
3. Cutlip DE, Chauhan MS, Baim DS et al. “Clinical restenosis after coronary stenting: Perspectives from multicentre clinical trials”. JACC, vol. 40, no 12, pp. 2082–2089, 2002.
4. (International Committee of Medical Journal Editors/no authors listed). Uniform requirements for manuscripts submitted to biomedical journals. Ann Intern Med, vol. 126, no 1, pp. 36–47, 1997.
5. Walker E, Nowacki AS. “Understanding Equivalence and Noninferiority Testing”. Journal of General Internal Medicine, vol. 26, no 2, pp.192–96, 2011.
6. Berman DS, Hachamovitch R, Shaw LJ, et al. “Roles of nuclear cardiology, cardiac computed tomography, and cardiac magnetic resonance: Noninvasive risk stratification and a conceptual framework for the selection of noninvasive imaging tests in patients with known or suspected coronary artery disease”. J Nucl Med, vol. 47, no 7, pp. 1107–18, 2006.
7. Shaw LJ, Wilson PW, Hachamovitch R, et al. “Improved near term coronary artery disease risk classification with gated stress myocardial perfusion SPECT”. JACC Cardiovasc Imaging, vol. 3, no 11, pp. 1139–48, 2010.
8. Shaw LJ, Berman DS, Maron DJ, et al. “Optimal medical therapy with or without percutaneous coronary intervention to reduce ischemic burden: Results from the Clinical Outcomes Utilizing Revascularization and Aggressive Drug Evaluation (COURAGE) trial nuclear substudy”. Circulation, vol. 117, no 10, pp. 1283–91, 2008.
9. Farzaneh-Far A, Phillips HR, Shaw LK, et al. “Ischemia change in stable coronary artery disease is an independent predictor of death and myocardial infarction”. JACC Cardiovasc Imaging, vol. 5, no7, pp. 715–24, 2012.
10. Juszczak E, Altman DG, Hopewell S, Schulz K. “Reporting of Multi-Arm Parallel-Group Randomized trials: Extension of the CONSORT 2020 Statement”. JAMA, vol. 321, no 16, pp. 1610–1620, 2019.

## Abbreviations

CABG: Coronary artery bypass grafting
DAPT: Dual antiplatelet therapy
FFR: Fractional flow reserve
HCR: Hybrid coronary revascularization
HT: Heart team
MACCE: Major adverse cardiac and cerebrovascular events (death/stroke/myocardial infarction (MI)/clinically-driven TVR)
MIDCAB: Minimally invasive direct coronary artery bypass
MV-CAD: Multivessel coronary artery disease
PCI: Percutaneous coronary intervention
RI: Residual myocardial ischemia
SPECT: Single-photon emission computed tomography
TVR: Target vessel revascularization
TVF: Target vessel failure.
